# The effect of different intracanal medicaments on the dislodgement resistance of mineral trioxide aggregate

**DOI:** 10.1186/s12903-022-02213-2

**Published:** 2022-05-25

**Authors:** Farzaneh Afkhami, Shahrzad Razavi, Sholeh Ghabraei

**Affiliations:** 1 Department of Endodontics, School of Dentistry, Tehran University of Medical Sciences, International Campus, Mahan St, Navab Highway, Tehran, Iran; 2 General Dentist, Tehran University of Medical Sciences, Tehran, Iran

**Keywords:** Bond strength, Calcium hydroxide, Intracanal medicaments, Mineral trioxide aggregate, Root canal disinfection, Nanoparticles

## Abstract

**Background:**

This study aimed to assess the effect of different commercially used calcium hydroxide [Ca(OH)_2_], the mixture of Ca(OH)_2_ + silver nanoparticles (AgNPs), and other intracanal medicaments on dislodgement resistance of mineral trioxide aggregate (MTA) to root canal dentin in short- and long-term application.

**Methods:**

Forty-six human single-rooted maxillary teeth were sectioned horizontally at mid-root into 1 mm thick slices. The lumen of the slices was standardized using #2–#5 Gates Glidden drills to a standard diameter of 1.3 mm in all samples. After smear layer removal, the samples were randomly divided into eight groups (n = 20) and treated with the following medicaments; Ca(OH)_2_ paste, Calcipex, Metapex, chlorhexidine (CHX), Ca(OH)_2_/CHX paste, Ca(OH)_2_/AgNPs, triple antibiotic paste and control group (normal saline). The samples were then incubated at 37 °C with 100% humidity for 1 week. Next, half of the teeth in each group (n = 10) were removed from the incubator and washed in an ultrasonic bath. ProRoot MTA was placed in the canal lumen and the samples were incubated at 37 °C and 100% humidity for 48 h to allow complete setting of MTA. The remaining half in each group (n = 10) was subjected to the same process after 1 month of application of medicaments. The dislodgement resistance of MTA to root dentin was measured. The data were analysed using two-way ANOVA and Tukey’s post hoc test.

**Results:**

No significant difference was noted in dislodgement resistance of samples after 1 week and 1 month in any group (P > 0.05). The only significant difference was noted between the control and CHX groups and the higher dislodgement resistance was recorded in the CHX group (P = 0.006). No other significant differences were noted between the groups (P > 0.05).

**Conclusion:**

Duration of application and the type of intracanal medicament do not affect the dislodgement resistance of MTA to root dentin. Although there was no statistically significant difference in the dislodgment resistance of MTA between the medicaments, CHX had a promising effect.

## Background

In order to achieve a relatively sterile canal system, chemo-mechanical disinfection is necessary, consisting of mechanical canal preparation and the use of intracanal medicaments [1]. Intracanal medicaments are known to considerably diminish the bacterial load and can be used in different conditions, such as multiple visit endodontics after trauma or in regenerative endodontics [2, 3].

Recently, triple antibiotic paste (TAP) has been introduced as an effective intracanal medicament in eliminating the bacterial flora. There are three antibiotics constituting the antibacterial paste: ciprofloxacin, metronidazole, and minocycline [4]. Among many applications of TAP, revascularization and regenerative treatment of immature teeth with open apexes are the most frequently used, offering biocompatibility and antimicrobial capacity, as well as the ability to disinfect root canal system [5]. Furthermore, apex formation and thickening of the dentinal walls are TAP advantages in regenerative treatment [6].

Chlorhexidine (CHX) with 2% concentration has been mentioned as an intracanal medicament substitute to calcium hydroxide [Ca(OH)_2_] with a lower toxicity and a wide range of antimicrobial effectiveness [7]. Some studies stated that in order to avoid some adverse effects of TAP like discoloration, antibiotic resistance and questionable biocompatibility of the medicament, CHX can be used in regenerative treatment without those unfavourable outcomes [6].

Ca(OH)_2_ has long been used for the elimination of pathogenic microorganisms from the root canal system and is among the most commonly used medicaments worldwide [3]. However, some studies failed to show the efficacy of Ca(OH)_2_ against all intracanal microorganisms [8–11]. Therefore some studies added antibacterial materials, such as silver nanoparticles (AgNPs), to the Ca(OH)_2_ and showed better antibacterial activity [8].

Nanotechnology is a recent approach widely used in many medical fields [12]. AgNPs are among the most desirable metals used in dentistry. They have the advantages of being small, inert nature as well as biocompatible and biologically safe [13]. Because of their antibacterial properties, AgNPs can be used as an intracanal medicament in dentistry [14]. In order to enhance Ca(OH)_2_ antibacterial feature, it is advisable to mix it with AgNPs which results in higher antibacterial properties at low concentrations with no obvious adverse effects on the other properties such as teeth discoloration [15, 16]. Many studies validated the fact that AgNPs/Ca(OH)_2_ combination can act effectively as an intracanal medicament due to the higher antibacterial feature [14, 15].

Mineral trioxide aggregate (MTA) is a bioactive substance with several applications such as perforation repair, apexification, coronal barrier formation and endodontic regenerative treatments [17, 18]. Physical and chemical properties of MTA are affected by several parameters such as surface properties of dentin, type of MTA [19], acidic environment [20], water to powder ratio [21], alkaline environment [17] and the ratio of propylene glycol to other constituents [22]. Moreover, surface modifications of dentin due to factors such as presence of smear layer [23], root canal irrigating solutions [24], laser irradiation [25], types of intracanal medicaments [3, 26] may affect the bond strength of dentin to MTA. Therefore, use of Ca(OH)_2_ and other intracanal medicaments may affect the bond strength of MTA to root dentin [17].

Adaptation and bond strength of endodontic materials to dentinal walls affect the leakage of bacteria and their products which is proved to be the main cause of endodontic failure. Therefore, adequate adaptation and optimal bond strength of materials to root canal walls are important [27].

Materials used in root canal treatment must be able to resist dislodging forces and maintain their bond to root canal dentinal walls when the tooth is in function or when other restorative materials are packed over them [27, 28]. The push-out test is routinely performed to test the bond strength of materials to dentinal walls [3, 19, 27, 28].

To the best of our knowledge, no previous study has compared the effect of AgNPs/Ca(OH)_2_ combination on dislodgement resistance of MTA to dentin. Thus, this study aimed to assess the effect of AgNPs/Ca(OH)_2_ combination and other commonly used intracanal medicaments as well as their short- and long-term application on bond strength of MTA to root canal dentinal walls. Moreover, the effect of different commercially used Ca(OH)_2_ medicaments was also investigated. The null hypothesis was that the use of different medicaments for short or long periods of time would have no effect on dislodgement resistance of MTA to root dentin.

## Methods

### Preparation of samples

The study protocol was approved by the Ethics Research Committee of Tehran University of Medical Sciences (Approval No: REC.1395.2358). Forty-six human single-rooted maxillary teeth freshly extracted due to periodontal or orthodontic reasons were used in this study. The teeth had straight roots without cracks, resorption or caries. The teeth were sectioned at the mid-root by a cutting machine (Delta, Tehran, Iran) and 160 slices, 1.00 ± 0.05 mm in thickness, were obtained. The lumen of the slices was standardized using #2 to #5 Gates Glidden drills to a standard diameter of 1.3 mm.

The smear layer was removed by immersion of the samples in 17% EDTA for three minutes, which was followed by immersion in 2.5% sodium hypochlorite in an ultrasonic bath for three minutes. Next, the samples were rinsed with water and dried. The slices were then randomly divided and the slices lumen was first filled and then immersed into the following medicaments listed as seen in Table 1.

**Table 1:** Preparation of mixtures and experimental groups

Groups	Medicaments	Composition/manufacturer
Ca(OH)_2_	Calcium hydroxide paste	Calcium hydroxide powder (i-dental, Šiauliai, Lithuania) mixed with normal saline with a ratio of 3:1
Calcipex	Calcium hydroxide paste	Calcipex II, Nishika, Japan
Metapex	Calcium hydroxide with iodoform	Metabiomed, Chungbuk, Korea
CHX	2% Chlorhexidine	Ultradent, Koln, Germany
Ca(OH)_2_/CHX	Calcium hydroxide and chlorhexidine paste	1:1 ratio; (Calcipex II, Nishika, Japan)/(Ultradent, Koln, Germany)
Ca(OH)_2_/AgNPs	Calcium hydroxide powder and silver nanoparticles suspension	Calcium hydroxide powder (i-dental, Šiauliai, Lithuania)/silver nanoparticles suspension (50 ppm, size ~ 20 nm) with a ratio of 3:1
TAP	Triple antibiotic paste	Metronidazole (Parsdarou, Tehran, Iran), Ciprofloxacin (Aria, Tehran, Iran), Minocycline (HEXAL AG, Holzkirchen, Germany) with a ratio of 1:1:1
NS	Control group	Normal Saline: 0.9% NaCl

The samples were incubated at 37 °C and 100% humidity for 1 week. Next, half the samples in each group were removed from the incubator and cleaned in an ultrasonic bath containing 10 mL of sodium hypochlorite and 10 mL of saline and dried. ProRoot MTA (Dentsply, York, USA) was mixed according to the manufacturer’s instructions and applied to the root canal space using a MTA carrier. The samples were then incubated at 37 °C and 100% humidity for 48 h to allow complete setting of MTA. The remaining half in each group was removed from the incubator after 1 month of application of medicaments and subjected to the same process.

A universal testing machine (Zwick Roell, Ulm, Germany) was used to assess the dislodgement resistance of MTA to dentin. The load was applied to MTA by a plugger with a 0.82 mm diameter at a crosshead speed of 1 mm/min. Load was applied in a coronal-apical direction until the MTA was detached from the dentin. Maximum load at failure was recorded. The Bond strength of each sample was calculated in MPa using the formula: Load applied in N/Area occupied by MTA. The latter was calculated using the formula: 2πrh, where π is a constant value (3.14), r: is the radius of each sample and h is the height of each sample.

**Fig. 1:**
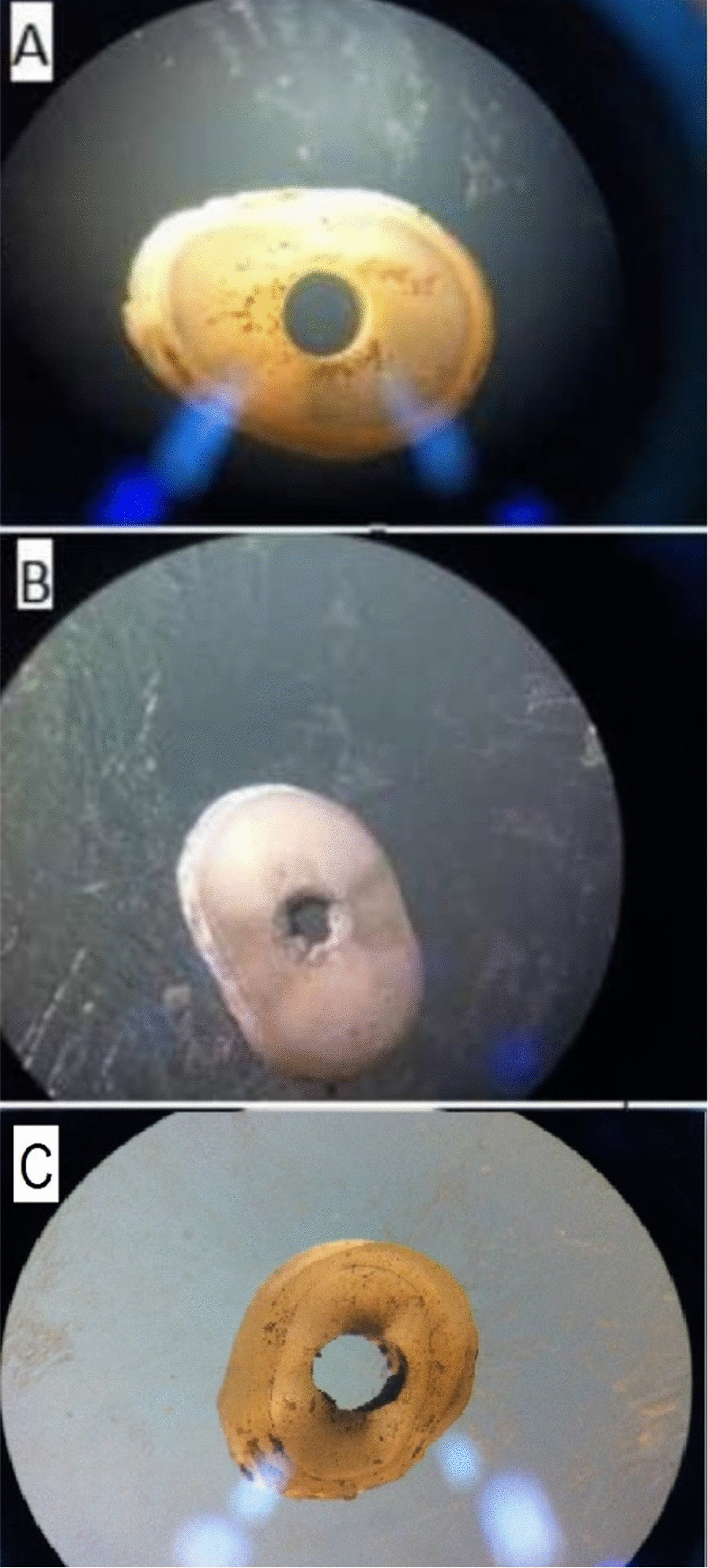
Inspection of the species under a stereomicroscope (× 40) **A**: Adhesive failure, **B**: cohesive failure, **C**: mixed failure

After completion of the push-out test, the samples were inspected under a stereomicroscope (Nikon, Tokyo, Japan) at × 40 magnifications to determine the mode of failure, which was categorized as cohesive, adhesive or mixed. Adhesive failure refers to fracture at the MTA-dentin interface. Cohesive failure refers to fracture within the MTA and mixed failure refers to a combination of adhesive and cohesive failures (Fig. 1).

The mean and standard deviation (SD) of dislodgement resistance in each group were calculated and the data were analysed using two-way ANOVA and Tukey’s post-hoc test. P < 0.05 was considered statistically significant.

## Results

The mean and SD of dislodgement resistance in the eight groups are shown in Table 2. The results of two-way ANOVA revealed no significant difference in the dislodgement resistance of the samples after 1 week and 1 month (P > 0.05). Tukey’s post hoc test revealed a significant difference in dislodgement resistance between the control and CHX groups (P = 0.006) and no other significant differences were noted between the groups (P > 0.05).

**Table 2:** The mean and standard deviation (SD) of the samples

Groups	Mean ± SD (MPa)		
	1 week (n = 10)	1 month (n = 10)	P value
Ca(OH)_2_	2.58 ± 1.56	2.13 ± 0.93	0.434
Calcipex	2.93 ± 1.97	1.75 ± 1.11	0.455
Metapex	2.45 ± 1.52	2.07 ± 1.72	0.575
CHX	3.08 ± 1.78	3.24 ± 1.92	0.006
Ca(OH)_2_/CHX	2.42 ± 0.99	2.82 ± 1.60	0.169
Ca(OH)_2_/AgNPs	2.56 ± 1.16	2.20 ± 1.99	0.402
TAP	2.03 ± 1.59	1.40 ± 0.77	0.996
NS	1.64 ± 0.70	1.11 ± 0.67	

No significant difference was noted between the experimental groups (P > 0.05). The only significant difference in dislodgement resistance was between the control (NS) and CHX groups (P = 0.006). The P values between the experimental groups and control group were shown in the table

The frequency of different modes of failure in the eight groups is shown in Table 3.

**Table 3:** The frequency of different modes of failure for each group

Groups	Time	n	Adhesive	Cohesive	Mixed
Ca(OH)_2_	1 week	10	4	2	4
	1 month	10	2	4	4
Calcipex	1 week	10	5	1	3
	1 month	10	5	0	5
Metapex	1 week	10	5	1	4
	1 month	10	4	1	5
CHX	1 week	10	6	1	3
	1 month	10	3	0	7
Ca(OH)_2_/CHX	1 week	10	2	1	7
	1 month	10	4	1	5
Ca(OH)_2_/AgNPs	1 week	10	4	2	4
	1 month	10	3	1	6
TAP	1 week	10	6	4	0
	1 month	10	7	2	1
NS	1 week	10	6	0	4
	1 month	10	3	1	6
Total (%)			43.12%	13.75%	43.12%

Adhesive and mix failures had the highest (43.12%) and cohesive failure had the lowest frequency (13.75%).

## Discussion

An ideal root canal filling material must be able to resist dislodging forces and mechanical stresses. The push-out test is performed to assess the dislodgement resistance of endodontic materials used for perforation repair or as a root-end filling material to ensure their efficacy [27].

MTA is considered a favorable endodontic material since it can prevent bacterial leakage, is stable against dislodging forces and provides a hermetic seal to dentinal walls [19]. However, the use of MTA for root perforation repair, in open apex necrotic teeth and in endodontic regenerative treatment often requires pre-treatment with intracanal medicaments. Application of intracanal medicaments may affect the sealing ability or adaptation of MTA to dentinal walls or its dislodgement resistance [17]. This study sought to assess the effect of different compositions of Ca(OH)_2_, CHX, TAP and mixture of Ca(OH)_2_/AgNPs on the dislodgement resistance of MTA to root dentin after 1 week and 1 month intervals. According to the above findings, the use of different testet medicaments in the short or long-term application would have no significant difference on dislodgement resistance of MTA to root dentin and the null hypothesis is accepted.

Nagas et al. [3] evaluated the bond strength of MTA and Biodentine to root dentin following 1 week of application of intracanal medicaments. They used three groups of Ca(OH)_2_ powder, TAP and combination of amoxicillin and clavulanic acid. They reported that Ca(OH)_2_ yielded the highest bond strength among the intracanal medicaments. However, in our study, different combinations of commercially available Ca(OH)_2_ and TAP had no significant differences with other groups in terms of dislodgement resistance. Such a controversy in the results of the two studies maybe due to different testing conditions and methodologies. The sectioning of the samples into slices of 1 mm thickness after the application of the medicaments used in Nagas et al. study could have an effect on the exposure of the dentinal walls to the intracanal medicaments. They sectioned the samples into 1 mm thick slices after the application of medicaments, while in the present study the samples were sectioned prior to the application of the medicaments allowing the slices to be more exposed to the intracanal medicaments.

Topçuoğlu et al. [27] evaluated the effect of Ca(OH)_2_ and several types of antibiotic pastes as intracanal medicaments on MTA bond to dentin. They tested several metronidazole and ciprofloxacin-based antibiotic pastes and showed no significant difference in bond strength of MTA between the two groups of control and TAP. Similarly in our study, no significant difference was noted between the control and TAP groups in terms of dislodgement resistance of MTA to root dentin. Sariyilmaz et al. [29] evaluated the effect of CHX and sodium hypochlorite as irrigants on the push-out bond strength of MTA. They concluded that CHX deteriorates the dislodgment resistance of MTA, whereas the present study demonstrates that CHX had effectively enhanced the push-out bond strength of MTA in comparison to the control group. This dissimilarity maybe due to the fact that in our experiment we first filled and immersed the tooth slices in the intracanal medicaments and then applied MTA contrary to the study of Sariyilmaz et al. in which the tooth slices were primarily filled with MTA and later immersed in irrigants. Tooth slices exposed to CHX prior to MTA application can alter dentinal structure and as a result improve MTA bond to dentine. A further factor which can modify the result of our study is the incubation time. We incubated the slices for 1 week and 1 month whereas in the study of Sariyilmaz et al. [29] the specimens were incubated only for 10 min.

Saghiri et al. [17] evaluated the bond strength of MTA in alkaline environment and showed that adhesive failure had the highest frequency. Shahi et al. [28] assessed the effect of different mixing techniques on the bond strength of white MTA, they both reported adhesive failure to have the highest frequency. Guneser et al. [24] studied the effect of different irrigating solutions on bond strength of Biodentine compared to restorative materials used for root perforation repair and showed that adhesive type was the most frequent mode of failure of MTA bond. Adhesive bond failure in the present study had the highest frequency similar to the results of the three aforementioned studies.

Topçuoğlu et al. [27] assessed the effects of Ca(OH)_2_, TAP plus minocycline, TAP plus cefaclor and double antibiotic paste (DAP) intracanal medicaments on bond strength of MTA and reported that cohesive failure of MTA was the most frequent in the Ca(OH)_2_ and TAP plus minocycline groups. Mixed failure had the highest frequency in the control group. However, in our study, mixed and adhesive failures had the highest frequency in the Ca(OH)_2_ powder and TAP groups respectively. The difference in the frequency of modes of failure between our study and that of Topçuoğlu et al. [27] maybe due to shorter setting time of MTA in our study (2 days) compared to the setting time in Topçuoğlu et al. study (4 days).

In another study, Guneser et al. [24] assessed the effect of different root canal irrigating solutions on the bond strength of Biodentine and MTA to root dentin. They reported that CHX decreased the bond strength of MTA to root dentin. In our study, CHX yielded the highest MTA bond to dentin, which is in contrast to the findings of Guneser et al. [24]. The difference in the results of the two studies maybe due to the duration of CHX application. On the other hand, it has been stated that due to its chemical properties, CHX reversibly bonds to pellicle on the root surface, hydroxyapatite and tooth [30]. Such a reversible bond by CHX may positively affect the bond strength of MTA to dentin surfaces.

Turk et al. [31], in their experiment on the effect of duration of application of intracanal medicaments on bond strength of MTA to dentin reported that bond strength of MTA decreased more significantly after 12 weeks of application of medicaments compared to 1 week, irrespective of the type of medicament. Based on their findings, TAP and double antibiotic paste decreased the bond strength of MTA after 2 weeks, while Ca(OH)_2_ powder had no effect on MTA bond strength after 2 and 4 weeks. The control medicament had no effect on bond strength at any tested time. Turk et al. concluded that both the type of medicament and its duration of application affect the bond strength of MTA to root dentin. In our study, different intracanal medicaments such as commercially available types of Ca(OH)_2_, CHX, Ca(OH)_2_/AgNPs and TAP were used for 1 week and 1 month. In contrast to Turk et al. our findings have shown that the duration of application of medicaments had no effect on MTA bond strength to root canal dentin.

Despite the benefits of using medicaments, complete removal of intracanal dressing materials is a concern, since it is crucial that MTA remains in place in the face of dislodging forces particularly when used for apexification or regenerative endodontics. No method has yet been demonstrated to fully remove intracanal medicaments from the root canal. In this case, there is a possibility that medicament residues may affect the adhesive strength of MTA to root canal dentine [3].

In the study by Turkaydin et al. [32] the removal of Ca(OH)_2_ and Ca(OH)_2_ with iodoform and p-chlorophenol paste (Calcipast Forte) was examined using EndoActivator, CanalBrush, and passive ultrasonic irrigation. They found removing Calcipast Forte more difficult than a water-based calcium hydroxide paste. A thorough removal of root canal medicaments was not achieved by any of the techniques evaluated in that study. Compared to Ca(OH)_2_, TAP residues appeared to have greater retention and deeper penetration; The Ca(OH)_2_ residues were rather superficial and contained a smaller amount of material. In the peresend study the mentioned medicaments had no effect on MTA bond strength to root canal dentin. The reason may be attributed to the application time. It was proved that dislocation resistance of MTA was significantly reduced by Ca(OH)_2_ after 12 weeks of treatment. However, short-term applications of Ca(OH)_2_ did not exhibit such an effect, thereby indicating that short-term Ca(OH)_2_ application is safe [31].

Rödig et al. [33] used simulated root canal irregularities to test the efficacy of three minutes of ultrasonic irrigation and RinsEndo in removing calcium hydroxide and Ledermix paste. It was found that none of the irrigation techniques succeeded in completely removing the intracanal medicaments from the apical part. The same result was achieved by Pabel and Hülsmann [34] when comparing the effectiveness of different techniques to remove Ca(OH)_2_ from straight root canals and found that no technique could achieve complete removal of calcium hydroxide from the root canal. In the study by Pabel and Hülsmann [34], they compared four different techniques for the removal of calcium hydroxide from straight root canals, although passive ultrasonic irrigation for 3 min resulted in the highest degree of cleanliness. In straight, wide root canals, 3 min of ultrasonic irrigation is more effective than syringe irrigation at removing artificially placed dentine debris [35]. Using ultrasonic agitation also helped remove intracanal medication more effectively than using the ProTaper rotary file and needle irrigation [36]. Therefore, based on the results of the aforementioned studies, we used ultrasonic bath to conduct ultrasonic agitation for 3 min on dentin slices in order to remove the medicaments as well as smear layer prior to medicaments application. The smear layer created by the filing of the canal walls causes intracanal medicaments residue to remain in the canal and makes their removal difficult by other means [3].

Although the present study did not aim to evaluate the effect of medicaments on dentin discoloration, based on our observation, TAP did have an impact on the discoloration of the samples. Even though the discoloration effect of TAP was not a major factor to discuss in our study but several literatures reported the same findings. AlSaeed et al. [37] detailed that TAP at high concentration (1 g/ml) caused a significant change in teeth color. Sabrah et al. [38] reported that due to the presence of minocycline in TAP, tooth colour change is inevitable in regeneration treatments. This undesirable effect was observed in the present study.

## Conclusion

With respect to limitations of this in-vitro study, it could be concluded that different types of commercially available Ca(OH)_2_ had no significant difference with each other in terms of their effect on MTA bond strength to root dentin. One week and 1 month application of intracanal medicaments had no significant effect on bond strength of MTA to root dentin. Irrespective of the duration of application, different types of tested canal medicaments had no significant differences with one another in terms of their effect on MTA bond strength to root dentin although CHX had a promising effect.

## Data Availability

Data available on request from the corresponding author.

## Abbreviations

MTA: Mineral trioxide aggregate
Ca(OH)_2_: Calcium hydroxide
CHX: Chlorhexidine
TAP: Triple antibiotic paste
ANOVA: Analysis of variance
